# Designing Coloring-Based Digital Art Therapy to Treat Alexithymia in Chinese College Students: Qualitative Study

**DOI:** 10.2196/82128

**Published:** 2026-06-01

**Authors:** Shuzhan Liu, Haoyong Deng, Jing Chen, Qi Wang

**Affiliations:** 1 College of Design and Innovation, Tongji University, Shanghai, China; 2 School of Nursing and Health Management, Shanghai University of Medicine and Health Sciences, No. 279 Zhouzhu Road, Pudong New Area, Shanghai, 201318, China, 86 13761489529

**Keywords:** alexithymia, digital art therapy, coloring game, intervention framework, emotional regulation, ER, semistructured interview, co-design

## Abstract

**Background:**

Alexithymia is a cognitive-emotional condition characterized by difficulties in identifying and expressing emotions. It lacks recognition among Chinese college students due to cultural norms that emphasize emotional restraint. Traditional interventions often rely on verbal expression, which poses challenges for students with alexithymia. Coloring-based digital art therapy provides a nonverbal, low-cognitive-load alternative that facilitates emotional expression through visual and interactive methods.

**Objective:**

This study aimed to explore expert opinions on developing a coloring-based digital art therapy intervention for alexithymia among Chinese college students, develop an intervention framework, and create a digital game prototype tailored to this population.

**Methods:**

This study was conducted in 2 phases. First, semistructured expert interviews were conducted with 10 experts to gather insights and create the intervention framework for coloring-based digital art therapy. In the second phase, a co-design process with the same experts refined the framework into a game prototype, with their feedback on design recommendations and game elements guiding the iterative development of *Coloring the Emoji*.

**Results:**

Experts identified four core design themes: (1) visual over verbal expression, (2) creating a natural and everyday experience, (3) supporting implicit expression, and (4) ensuring psychological safety and data protection. These principles were incorporated into *Coloring the Emoji*, which guides students through color selection, emoji-based pattern selection, and free drawing to promote emotional expression. Key features of the game include the use of familiar emojis, customizable options for emotional expression, and robust privacy protections. The game is freely accessible and is well-suited for Chinese college students dealing with academic pressures and financial constraints.

**Conclusions:**

This study presents a novel intervention framework for coloring-based digital art therapy tailored to Chinese college students with alexithymia. The game *Coloring the Emoji* provides a nonverbal, intuitive platform for emotional regulation and self-reflection, addressing the cultural and emotional challenges specific to this population. Future research should focus on user testing and explore its applicability to other cultural and age groups.

## Introduction

### Background

Alexithymia is a cognitive-emotional condition [1] that affects about 10% of the general population and more than 20% of college students [2]. It results from disrupted brain circuitry in emotional processing and is characterized by deficits such as difficulty distinguishing between emotions and bodily sensations, a lack of emotional vocabulary, and reduced symbolic imagination [3]. Our previous survey of Chinese college students (n=332) revealed that 12.05% of students exhibited significant alexithymia (20-item Toronto Alexithymia Scale ≥70), with an overall average score of 59.90 (SD 8.15), which is close to the clinical threshold, suggesting a significant prevalence of alexithymia in this population.

Existing interventions primarily rely on psychotherapy approaches such as cognitive behavioral therapy (CBT) and mindfulness-based stress reduction, which aim to alleviate symptoms by enhancing emotional awareness and expression [4]. These interventions typically require college students to verbally describe their emotions and engage in self-reflective emotional processing, which can be particularly challenging for college students with alexithymia due to their limited ability to identify emotions and express them verbally [3]. In China, where emotional restraint is culturally valued [5], reluctance to express emotions is often seen as normal social behavior rather than a clinical symptom [5], resulting in a lack of recognition of alexithymia and insufficient focus on tailored therapeutic approaches. Chinese college students face additional emotional challenges due to cultural expectations that prioritize emotional control and social conformity, making emotional expression more difficult [6]. Furthermore, practical barriers such as heavy academic workloads, geographic limitations, and financial constraints further hinder access to therapy, complicating the identification and treatment of alexithymia in this population [7,8].

### Art Therapy and Its Limitations

Art therapy offers a structured and theoretically grounded approach to addressing alexithymia [9]. By involving nonverbal creative media, such as color, imagery, and movement, art therapy facilitates emotional identification and expression in a psychologically safe and cognitively manageable way [1]. This process can enhance emotional awareness, reduce avoidance, and externalize implicit emotions [10], thereby supporting long-term self-understanding and emotional regulation. By facilitating the externalization of suppressed emotions and overcoming verbal barriers, art therapy alleviates symptoms of alexithymia [11], reduces anxiety and depression [12], and enhances overall well-being [13].

However, several significant limitations continue to constrain their broader applicability. First, attitudinal barriers, such as shame and embarrassment, may hinder full engagement [14], especially among Chinese college students, for whom emotional restraint is culturally valued, and open emotional expression is often seen as a weakness [6]. Second, a lack of initiative, often driven by emotional avoidance and suppression, impedes their ability to regulate and express emotions effectively [15]. Chinese students are often taught to suppress emotions in favor of academic and social expectations, making emotional engagement more challenging. Third, time constraints, spatial limitations, and financial barriers reduce the feasibility and accessibility of interventions [16]. Chinese college students face heavy academic workloads, which limit their time for therapy. Additionally, geographic barriers and limited financial resources make it difficult for students to consistently participate in traditional therapy.

To address these challenges, digital art therapy has emerged as a promising intervention that leverages technology to enhance emotional expression and psychological healing [17]. Compared to traditional methods, it offers greater accessibility, continuity of care, adaptability for college students, and expanded modes of self-expression.

### Coloring-Based Digital Art Therapy

Digital art therapy encompasses various approaches that combine creative expression with therapeutic intent. Among these, virtual reality–based art therapy offers an immersive, gravity-free environment that encourages full-body engagement and provides users with a sense of control. However, it requires costly equipment and may cause sensory discomfort, limiting its practical applicability, particularly for college students with limited finances [18]. Augmented reality–enhanced therapy, which overlays digital images onto real-world settings, is beneficial for trauma exposure and social interaction but poses challenges related to technical requirements and privacy concerns, which can hinder its widespread use [19].

Digital drawing platforms, such as Procreate or artificial intelligence–assisted tools, provide a low-pressure environment for precise, reversible emotional expression. However, they lack therapeutic framing and professional guidance, which can limit their effectiveness in addressing emotional regulation needs [20]. Therapeutic mobile apps offer a more structured approach, typically integrating mood tracking, daily prompts, and simple creative tasks. These apps are particularly accessible and well-suited for daily use, especially for college students dealing with emotional regulation challenges. However, they generally lack therapist involvement and offer limited depth of engagement, which may reduce their therapeutic effects [21]. Remote art therapy, delivered through live or asynchronous interaction, provides professional guidance and contextual sensitivity but may be hindered by technical disruptions and reduced nonverbal cues [22].

In this context, coloring-based digital art therapy builds on the accessibility and user-friendliness of digital platforms while offering unique advantages [23]. Unlike general digital drawing platforms, it integrates therapeutic guidance into its design by focusing on nonverbal emotional expression through color selection, pattern selection, and simple painting tasks [24]. This approach not only reduces cognitive load but also provides structured emotional support via predesigned templates, emotion tracking, reflective journals, and optional asynchronous therapist feedback [23]. By blending creative expression with therapeutic intent, coloring-based digital art therapy provides a safe, nonjudgmental space for users, making it particularly suitable for Chinese college students with alexithymia [25]. It helps them cope with the challenges posed by cultural emotional restraint and enables emotional exploration in a supportive environment [26].

### Serious Games in Digital Art Therapy

Serious games are widely used in therapeutic contexts, designed to achieve specific goals such as emotional regulation, behavior change, or psychological healing, while also engaging users with interactive and enjoyable gameplay [27]. These games are different from traditional entertainment games, as they balance 2 key elements: achieving therapeutic outcomes and maintaining user engagement through fun and immersive experiences [28]. This combination makes serious games effective in offering users an interactive platform for emotional expression and self-reflection, which is especially beneficial for individuals with alexithymia, who may find verbal expression challenging [29]. By incorporating structured tasks and feedback, serious games enable users to process and express emotions in a nonthreatening, low-pressure environment [27].

Among various serious games, coloring-based games stand out because of their simplicity and accessibility. These games create a low-cognitive-load environment for nonverbal emotional expression, allowing users to engage in activities such as color selection, pattern selection, and painting [24]. These tasks are specifically designed to help users externalize their emotions without the need for verbal expression, making them particularly useful for individuals with alexithymia [30]. In the case of Chinese college students, these games are especially beneficial as they provide a safe, nonjudgmental space for emotional exploration, which is crucial in a culture where emotional restraint is valued and emotional expression may be suppressed [31]. The game’s structure, which involves tasks such as selecting colors, choosing patterns, and free drawing, offers a broad scope for users to express emotions creatively [32,33]. Additionally, the game settings—including visual style, real-time feedback, customizable options, and privacy measures—ensure that the intervention is engaging, accessible, and culturally sensitive [34]. These elements create a therapeutic yet enjoyable experience that respects cultural norms and encourages students to express themselves in a way that feels both safe and meaningful [35]. By integrating creative expression with therapeutic support, coloring games offer a comprehensive, user-friendly intervention that promotes emotional regulation, self-reflection, and personal growth [36].

### Objectives

This study examines the use of coloring-based digital art therapy as an intervention for Chinese college students with alexithymia, focusing on how low-cognitive-load digital coloring tasks can support emotional identification and expression. The research began with expert interviews from fields including art therapy, human-centered design, digital health, and software development. These interviews provided valuable insights, which were used to create an intervention framework specifically tailored to the emotional and expressive needs of Chinese college students with alexithymia. Following the development of the framework, we engaged in a co-design phase with the same experts to refine and adapt the framework into a practical prototype. This iterative process ensured that the final game design, *Coloring the Emoji*, met both design recommendations from the previous framework and the specific needs of Chinese college students. The game, which incorporates color theory, pattern design principles, and human-computer interaction concepts, aims to facilitate nonverbal emotional exploration through intuitive color and pattern interactions. Future work will involve user testing to assess its therapeutic impact and further refine the system for broader application.

In summary, our contributions can be summarized as follows:

- An intervention framework for coloring-based digital art therapy was proposed to help Chinese college students with alexithymia better identify and describe their vague emotions, as well as effectively express and process them.
- Based on the proposed intervention framework, the authors collaborated with experts during a co-design phase to develop and refine *Coloring the Emoji*, a digital coloring game specifically designed to address the emotional needs of Chinese college students with alexithymia.

## Methods

### Overview

This study was conducted in 2 phases to construct the framework of coloring-based digital art therapy and to develop a coloring game for Chinese college students with alexithymia. In the first phase, semistructured expert interviews were conducted to gather professional insights and create an intervention framework for coloring-based digital art therapy. This qualitative approach helped identify key design principles and therapeutic mechanisms for the coloring game. In the second phase, we collaborated with the same experts in a co-design process to refine the framework and transform it into a game prototype. Through a series of collaborative discussions, the experts provided feedback on design recommendations and key game elements, allowing for the iterative development of *Coloring the Emoji*.

### Recruitment

Purposive sampling was conducted until thematic saturation was achieved. Participants were selected through the authors’ professional networks, targeting both professional experts and graduate students with relevant expertise in art therapy, digital health, human-centered design, or mobile app development. Professional experts were recruited for their extensive experience, with a minimum of 5 years in the field, as their expertise offers deeper insights and a broader understanding of the subject matter. Graduate students were chosen based on having over 5 years of professional experience in their respective fields and having participated in multiple practical projects relevant to the research topic. These graduate students were selected for their ability to provide insights closely reflecting real-world user experiences, given their engagement in daily life environments. To ensure diversity, experts were recruited from various regions of China. In addition to experts from the researchers’ base in Shanghai, representing the eastern region, experts were also sought from the northern and southwestern regions to capture the regional and cultural differences across the country.

As data collection progressed, the research team continuously analyzed the interviews to assess thematic redundancy and determine when saturation had been reached. Based on this criterion, a total of 10 participants (4 graduate students and 6 professional experts) were included in the study. The inclusion criteria were as follows: (1) a master’s degree or higher; (2) a minimum of 5 years of experience in art therapy, digital health, human-centered design, or mobile app development; and (3) demonstrated enthusiasm and willingness to participate. Exclusion criteria included experts who reported limited familiarity with the relevant fields or who provided low-quality responses during the initial consultation.

### Procedure

We arranged semistructured interviews with each expert between April 2024 and June 2024, coordinating the time and location in advance via email, phone, or text message. Due to geographical constraints, 2 experts were interviewed remotely via Zoom (Zoom Communications, Inc), while the remaining 8 experts were interviewed face-to-face by 2 members of the research team in a private setting. A semistructured interview guide with open-ended questions was prepared to allow each expert to share their perspectives and recommendations regarding coloring-based digital art therapy. The interview guide (Multimedia Appendix 1) was developed with 17 questions and addressed the following themes: (1) experiences in art therapy, (2) attitude toward digital art therapy, (3) suggestions for coloring-based digital art therapy, and (4) willingness of the expert to facilitate and support the follow-up study. Each interview lasted between 28 and 90 minutes. All interviews were audio- and video-recorded and verbatim transcribed within 3 days of each interview.

After the interviews, from June 2024 to July 2024, we proceeded to the second phase, engaging the same experts in multiple co-design sessions. These sessions were held remotely via Zoom and the Figma platform to refine the intervention framework and translate it into a game prototype. Experts provided feedback on the categorization of design recommendations and key game elements, ensuring the final design aligned with previous insights and therapeutic goals.

### Data Analysis

Data collected from the semistructured expert interviews were analyzed using NVivo 15 (Lumivero) and an inductive approach by 2 independent researchers who were part of the research team, identifying themes related to experts’ views on using digital art therapy for emotion regulation and their opinions on designing color-based interventions for Chinese college students with alexithymia. Each researcher independently reviewed the transcripts, coded the insights, and organized these codes into themes. The researchers then discussed their findings to reach a consensus on the key themes, ensuring agreement on the categorization of the data.

### Ethical Considerations

The study was conducted following ethical standards and was approved by the institutional review board of the main researchers’ affiliated institution (approval: TJDXSR076). Before participation, experts were informed of the study’s purpose, procedures, and potential risks. Verbal informed consent was obtained when scheduling the interview via phone or email. All participants took part in this research voluntarily without any financial compensation. All participants were assured of their confidentiality, and data were anonymized to prevent identification. Personal information was securely stored and anonymized throughout the study, ensuring compliance with data protection regulations.

## Results

### Expert Characteristics

A total of 10 participants were involved in the study, comprising 6 professional experts and 4 graduate students with relevant expertise. The gender distribution was balanced, with 5 males and 5 females. The graduate students, aged between 24 and 27 years, included 2 students aged 24 years and 2 students aged between 25 and 27 years. All were master’s degree students who had studied in their respective fields for 5 to 8 years. Their areas of expertise included art therapy (n=1), digital health (n=1), human-centered design (n=1), and mobile app development (n=1), with the students in art therapy and digital health each having more than 1 year of work experience. The experts ranged in age from 28 to 44 years, with 2 experts aged under 34 years and 4 experts aged between 34 and 44 years. All 6 experts held doctoral degrees, including 4 associate professors and 2 lecturers. The experts’ years of industry experience varied: 2 experts had nearly 10 years, 3 had nearly 20 years, and 1 had more than 20 years. Their areas of expertise included art therapy (n=1), digital health (n=2), human-centered design (n=2), and mobile app development (n=1). An overview of the expert characteristics is provided in Table 1.

**Table 1.** Basic information about consulting experts (N=10).

Characteristics and classification	Participants, n
Age (y)	
24	2
34	4
44	4
Professional title	
Associate professor	4
Lecturer	2
N/A^a^	4
Education background	
Doctoral degree	6
Master’s degree	4
Years of study	
5	4
Years of work	
10	2
15	1
20	3
Specialist areas	
Art therapy	2
Digital health	3
Human-centered design	3
Mobile app development	2

a Not available.

### Intervention Framework for Coloring-Based Digital Art Therapy

The thematic analysis of expert interviews provides valuable insights into the construct of coloring-based digital art therapy for addressing alexithymia among Chinese college students.

#### Theme 1: Visual Instead of Verbal

##### Subtheme: Draw the Emotions

Most experts (7/10) believed that art therapy plays a significant role in the treatment of emotional expression disorders. They pointed out that art therapy not only helps visualize abstract emotions but also eliminates language barriers through visual expression, providing college students with a low-pressure, nonthreatening way to externalize their emotions. A smaller group (3/10) mentioned more detailed points. They expressed that color makes the process more tangible and perceptible, promoting emotional regulation and self-awareness, and helping students more intuitively recognize and process their feelings. One expert mentioned that one of her college students used warm yellow to express maternal love and the warmth of ginger soup, “As I observed the boy’s coloring, he colored the ‘umbrella in the rain’ warm yellow, saying, ‘This is the steam from my mom’s ginger soup’” [E1].

Another expert also pointed out similar cases involving a student who used an orange boat to symbolize secret affection, “In one of my cases, … the student painted gray waves for 3 days, then suddenly added an orange boat, saying, ‘That’s the color of the headband of the girl I secretly like’” [E7].

##### Subtheme: Express Through Symbolism

Some experts (4/10) suggested that symbolic emotional expression is not limited to color alone, but also includes shapes, sounds, and other sensory elements, which can help college students more deeply recognize and communicate their emotions. Integrating various sensory symbols, such as visual and auditory cues, can make emotional expression more concrete and diversified. By adapting to different college students’ preferences, it breaks the limitations of traditional verbal expression. One expert stated, “When the student exhibited anger, the system responded with fast music and flames appeared around the character” [E6].

##### Subtheme: Describe Emotional Stories in the Third Person

Experts who have had long-term communication with college students (5/10) have found that by attributing emotions to virtual characters or a third party, college students can avoid directly confronting their own emotions, which makes it easier for them to express and understand their feelings. This method provides an indirect way to externalize emotions, making emotional expression more natural and less stressful:

*Asking “What does the person in the picture see?” works much better than asking “How do you feel?”*

[E1]

*Every time I asked about her feelings, the girl stubbornly said, “I feel nothing,” but when her cloud avatar turned gray, the system said, “Little Cloud is raining,” and she cried, “It’s because I want to cry.”*

[E9]

##### Subtheme: Provide Interactive Options

Experts with hands-on therapeutic experience (3/10) noted that body language, especially tactile and auditory feedback, can make emotional expression more concrete and vivid. By activating the body’s instinctive responses, college students can perceive and express emotions more intuitively. This method is beneficial for those who struggle with expressing emotions, as body language provides an effective alternative, such as adjusting brush size or the intensity of movements. This sensory experience makes emotional externalization more natural and enhances the emotional regulation process:

*A little girl I once treated expressed, “When I’m happy, I use a small brush to paint carefully, but when I’m unhappy, I use a thick brush.” So, it would be better to give them different choices.*

[E1]

*It would be beneficial to incorporate some interactive features, such as adding sound effects when the student clicks a button.*

[E8]

### Theme 2: Create a Natural and Everyday Experience

#### Subtheme: Refuse to Judge

Most experts (9/10) stressed the importance of avoiding the judgment of emotional expressions, as this could lead to students feeling evaluated or restricted in their emotional expression. When students are aware that their work will be graded or categorized according to predefined emotional labels, it introduces an element of judgment, which can hinder their ability to freely express their emotions:

*Core rule: No grading! Do not label the patient’s work. Grading and labeling will make students feel that they are being evaluated, which will affect their emotional expression.*

[E1]

*Students should have the freedom to give meaning to their own emotional expressions without having fixed labels imposed by the system or the outside world.*

[E5]

#### Subtheme: Integrate Into Life Scenes

In addition, experts (7/10) believe that the design of digital art therapy should integrate emotional expression into daily life to make it closer to students’ real experiences. By combining the stress and emotions of life with artistic expression, students can release their inner emotions in a familiar situation.

*When students face exam anxiety, some test paper-style patterns can be added to the system to help students vent their emotions through artistic expression when facing pressure and reduce the burden of stress.*

[E7]

#### Subtheme: Establish Event Connection

More than half of the experts (6/10) mentioned that through contextual connection, students can more naturally connect emotions with specific events, which helps them understand and express emotions more easily. The design of contextual connection can provide a guide to enable students to connect emotions with real-life events and situations, making it easier for them to identify and express emotions:

*Seeing the sunset makes the child calm, and the system learns to associate sunsets with relaxation.*

[E2]

*After watching her favorite flowers, the system links happiness with similar content.*

[E3]

#### Subtheme: Leverage Digital Technology

Experts (3/10) emphasized that the application of digital technology can further enhance the effectiveness of digital art therapy. By using modern digital tools, students can enjoy a more fluid, intuitive, and multisensory experience of emotional expression. One expert stated, “Quick responses and smooth experiences can reduce students’ anxiety caused by technical issues and help them focus on emotional expression rather than technical distractions” (E10).

### Theme 3: Support Implicit Expression

#### Subtheme: Anonymization

Experts (4/10) emphasized that anonymity is a key feature of digital art therapy, helping students feel safe and unjudged when expressing emotions. Anonymizing the process of emotional expression allows students to explore their feelings without fear of exposure or social judgment. This feature is especially beneficial for those who experience social anxiety or feel vulnerable when discussing their emotions openly. One expert stated, “Works should provide an anonymous upload option while retaining the students’ right to refuse uploading” (E10).

#### Subtheme: Protect Personal Privacy

Experts with a technical background (3/10) pointed out that privacy protection is another crucial issue in digital arts therapy and highlighted several methods for protecting personal data and ensuring the confidentiality of students’ emotional expressions. Protecting privacy is especially important when dealing with sensitive topics such as emotional distress, mental health issues, or trauma. Students need to be assured that their personal information and emotional content will not be exposed to others without their consent:

*Implement strict controls on who can access user data and when, ensuring only authorized people or systems can access sensitive data.*

[E3]

*Using a WeChat mini-program and bypassing campus network restrictions ensures that students can continue their coloring even if the internet is down at night.*

[E6]

### Theme 4: Ensure Psychological Safety and Data Protection

#### Subtheme: Protect User Data

Experts (10/10) agreed that data protection is a fundamental component of any digital therapy platform. Ensuring that students’ personal information and emotional data are securely handled and erased when no longer needed is essential for maintaining trust and preventing exploitation. One expert stated, “When deleting data, provide a destruction certificate to ensure complete disappearance” (E6).

#### Subtheme: Focus on Psychological Safety

In addition to data protection, experts (7/10) emphasized that the game’s design should prioritize mental safety. Ensuring that students feel emotionally safe, supported, and free from stress during the emotional expression process is essential. The design should minimize factors that could lead to psychological harm or cause unnecessary anxiety.

*Many college students I met expressed concerns that they could not draw, had no artistic talent, and were afraid that their paintings would not look good when they first encountered coloring art therapy. Psychological counselors and digital systems should always support and protect college students’ fragile hearts, encourage college students to try, and avoid any content that would frustrate college students’ psychology as much as possible.*

[E1]

*…Such as detecting repeated modifications triggers a pop-up: “Do you want to take a break?”*

[E3]

Based on the qualitative analysis of expert interviews, the intervention framework for coloring-based digital art therapy is summarized in Table 2.

**Table 2.** Intervention framework for coloring-based digital art therapy.

Themes and subthemes	Design recommendation
Visual instead of verbal	
Draw the emotions	Use color to externalize emotions by allowing students to select or create colors that represent different feelings or emotional states. This can be applied through various elements in the game, such as backgrounds, objects, or avatars.
Express through symbolism	Enhance emotional expression with not only color but also shapes, sounds, and sensory elements, offering a more diversified and personalized way to communicate emotions.
Describe emotional stories in the third person	Attribute emotions to virtual characters or third parties. Allow students to avoid confronting their own feelings directly, encouraging more natural, indirect emotional externalization.
Provide interactive options	Allow students to adjust the size, intensity, or speed of brush strokes, gestures, or movements. Implement vibration or sound effects that correlate with the movement or emotional expression, such as changes in the brush pressure or feedback sounds based on actions.
Create a natural and everyday experience	
Refuse to judge	Avoid grading or labeling emotional expressions.
Integrate into life scenes	Connect emotional expression to daily life experiences. Simulate real-life situations through themed templates or prompts that mirror common emotional triggers.
Establish event connection	Use contextual connections (like prompts, scenarios, or selectable environmental symbols) to link emotions with specific real-life events. Design the system to associate emotions with familiar triggers.
Leverage digital technology	Use digital technology to create a smooth, intuitive, and multisensory emotional expression experience. Ensure quick responses and smooth interactions.
Support implicit expression	
Anonymization	Allows students to express emotions freely without fear of judgment or exposure through anonymity. Provide an anonymous upload option while respecting the students’ right to refuse sharing their work.
Protect personal privacy	Implement strict access controls to ensure that only authorized people or systems can access sensitive user data. Provide offline functionality, such as bypassing network restrictions, to ensure students can continue their emotional expression work securely, even without internet access.
Ensure psychological safety and data protection	
Protect user data	Provide a data destruction certificate when deleting user information to ensure complete and irreversible removal.
Focus on psychological safety	Prioritize psychological safety by creating a supportive and stress-free environment. Implement gentle reminders or breaks to avoid overwhelming students.

### Coloring Game: *Coloring the Emoji*

**Figure 1.**
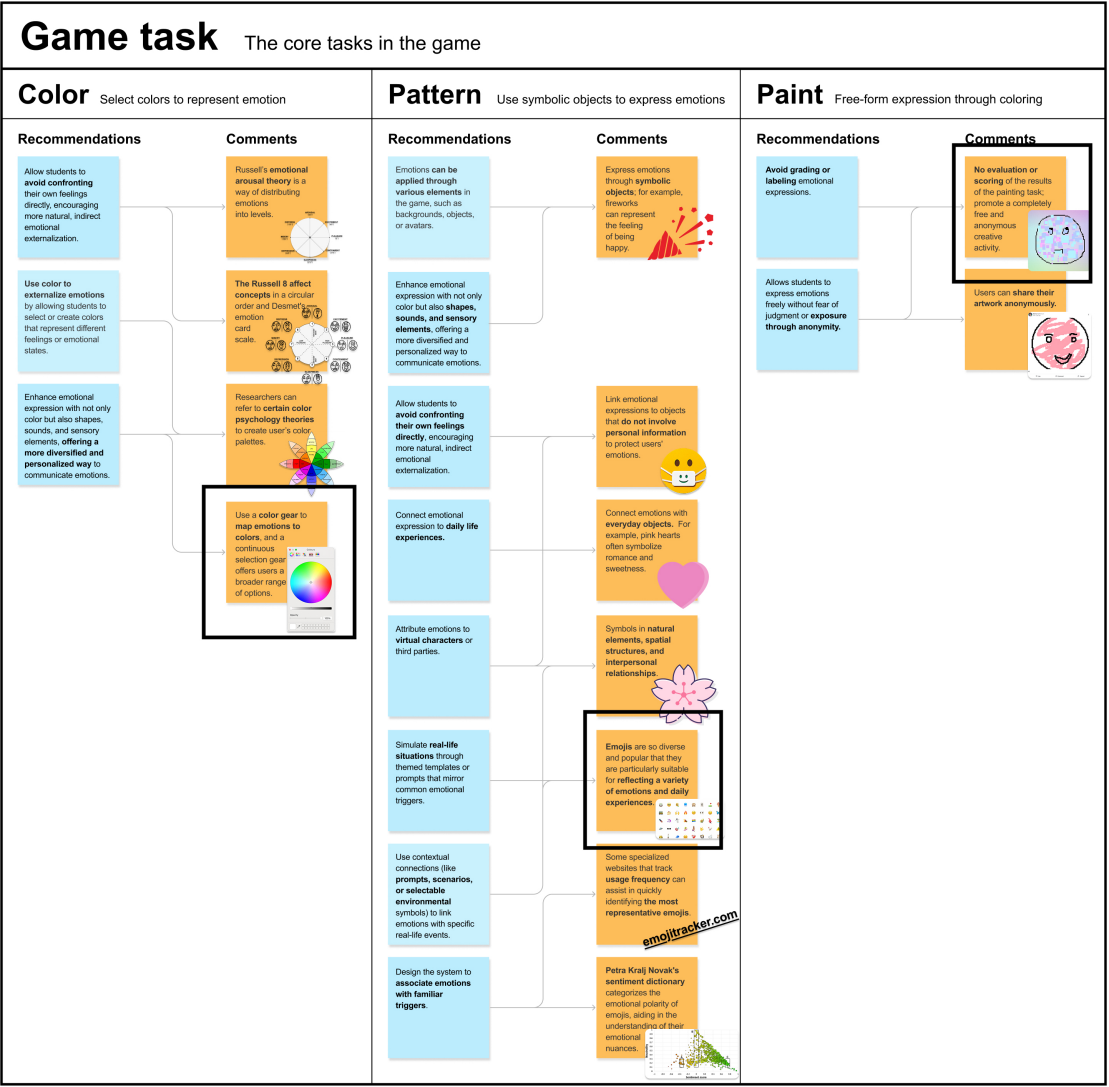
Game task.

**Figure 2.**
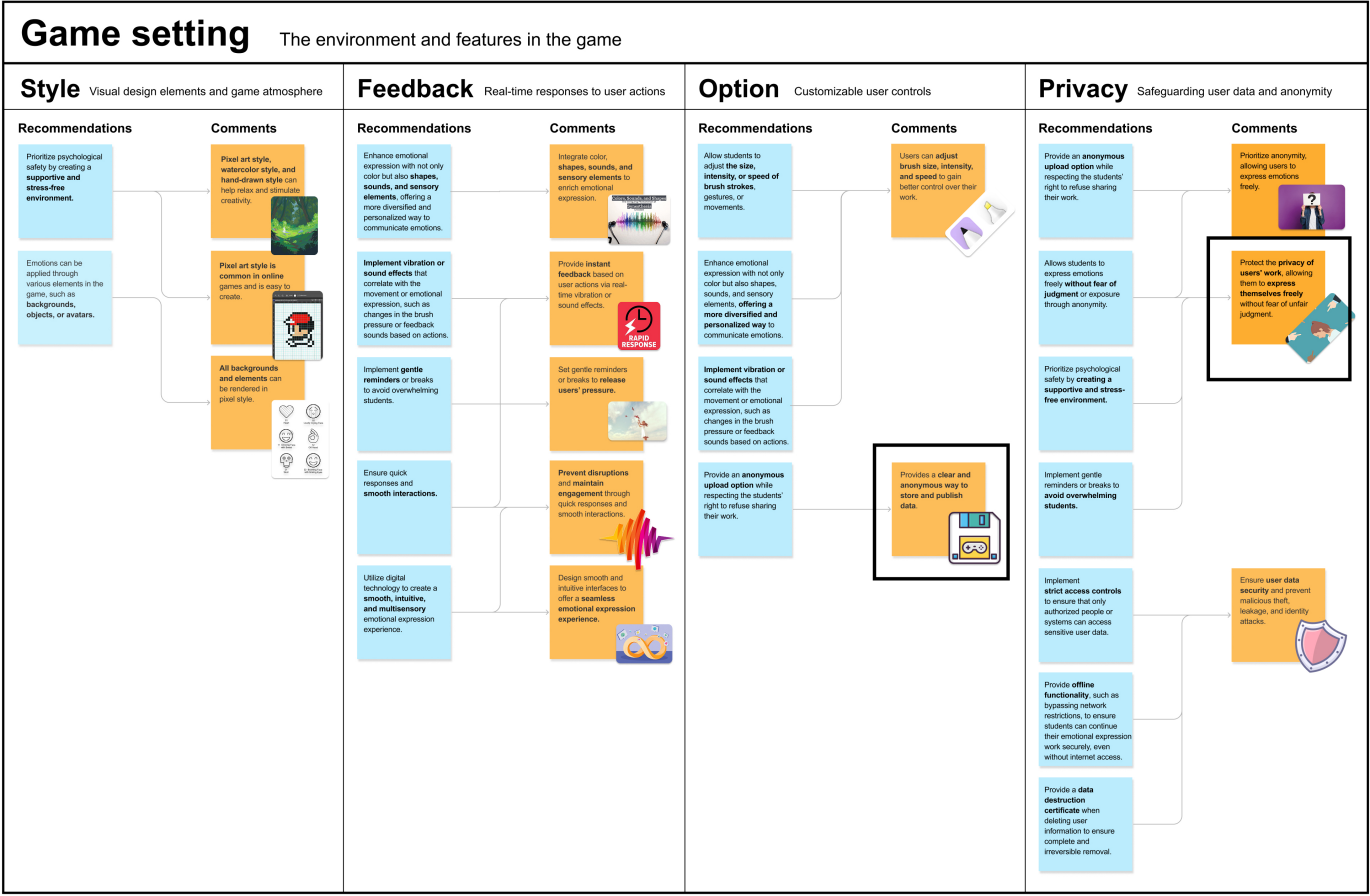
Game setting.

Based on the insights gathered from the co-design process, *Coloring the Emoji* was developed to address the emotional and cognitive challenges faced by Chinese college students with alexithymia. During co-design sessions, experts provided feedback on how to organize and apply the design recommendations, which led to the refinement of key game components, ensuring alignment with therapeutic goals (Figure 1 and Figure 2). The game was designed to facilitate intuitive, nonverbal emotional expression and recognition, incorporating expert insights into its core elements.

The game task includes 3 key components: color, pattern, and paint [32,33]. For color, experts recommended using color to externalize emotions, allowing students to select colors that represent different feelings. Experts also suggested incorporating a continuous color selection gear to offer a broader range of options for more personalized emotional expression. This led to the creation of the *Emotional Color Gear*, which maps emotions to colors based on Russell’s [37] emotional arousal theory and Desmet’s [38] emotion card scale, simplifying emotions into 2 dimensions—pleasure and arousal—for easier emotional identification. In pattern, experts emphasized the use of symbolic objects and familiar symbols to help students externalize emotions indirectly. This led to the development of the *Emoji Scene Library*, which includes 53 emojis with strong emotional polarity and the highest use frequency among college students [39,40], allowing students to connect their emotions to familiar symbols and everyday experiences. The system was designed to associate emotions with common triggers, facilitating easier emotional identification [41]. For paint, experts highlighted the importance of creating a nonjudgmental space for emotional expression, with no grading or labeling of expressions, and features enabling students to share their artwork anonymously, promoting free, creative activities.

The game setting includes 4 key components: style, feedback, option, and privacy [34,35]. For style, experts recommended creating a supportive and low-pressure environment through visual elements that could help students relax and stimulate creativity. Based on this, we chose the pixel art style, which is common in online games, easy to create, and helps to create a relaxed atmosphere. In feedback, experts emphasized enhancing emotional expression not only with color but also with shapes, sounds, and sensory elements to provide instant feedback, while introducing gentle reminders and breaks to avoid overwhelming students and maintaining their engagement. For option, experts recommended allowing students to adjust brush size, intensity, and speed to better control their emotional expression, thereby enhancing engagement [42]. In privacy, experts stressed anonymity and data security, implementing strict access controls and offering the option to share artwork anonymously. Additionally, the game includes a data destruction certificate to ensure the irreversible deletion of personal data.

To ensure *Coloring the Emoji* meets the specific needs of Chinese college students with alexithymia, several design features were integrated based on expert feedback. These features address challenges such as emotional restraint, heavy academic workloads, and financial limitations. First, to accommodate emotional expression, the *Emotional Color Gear* offers a continuous selection option, allowing a broader range of color choices to represent emotions. Second, considering the popularity of emojis in Chinese digital communication, a diverse set of emojis was incorporated, reflecting a wide range of emotions and linking them to daily life experiences. Third, the game avoids grading or scoring emotional expressions, aligning with the cultural preference for privacy and reducing social pressure. Finally, robust privacy protection measures ensure students can express themselves freely without fear of judgment, with the option to share artwork anonymously. These design choices address the unique emotional challenges faced by Chinese college students, offering a culturally sensitive space for emotional exploration and self-expression.

**Figure 3.**
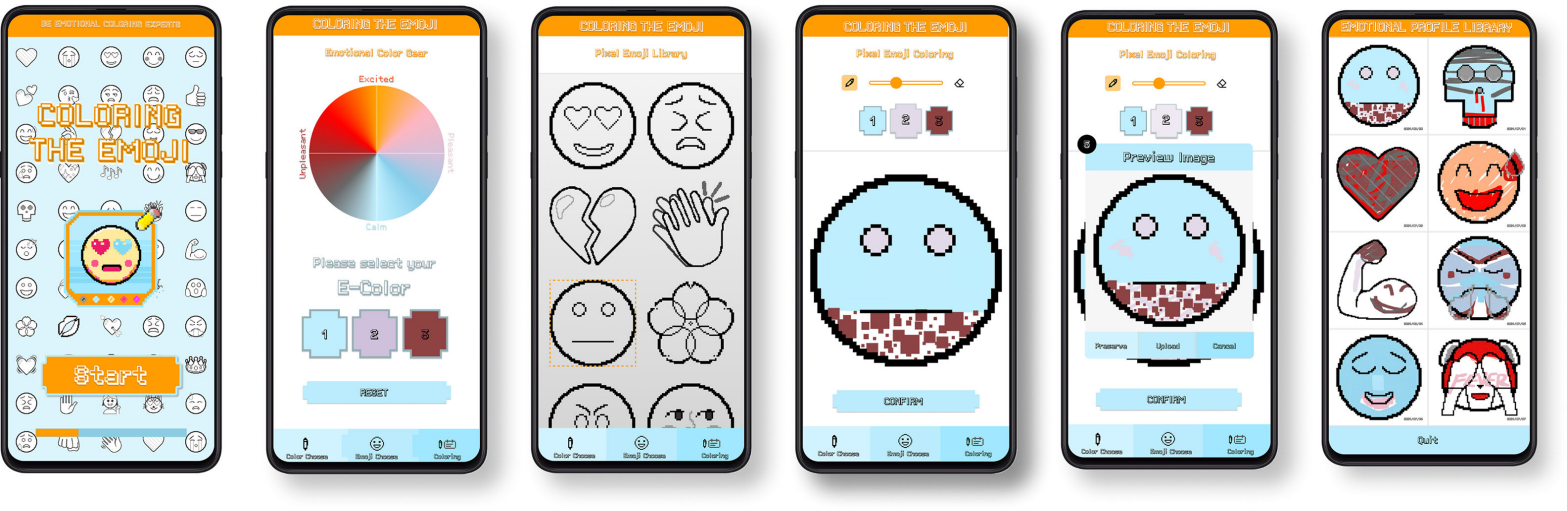
The core interface of *Coloring the Emoji*.

*Coloring the Emoji* helps college students with alexithymia recognize and express their emotions through 3 core stages: color selection, pattern selection, and free drawing (Figure 3). In the color selection stage, students select colors to externalize and convey their feelings, thereby clearly identifying their emotions. In the pattern selection stage, students choose emojis that represent their emotions, allowing them to express their feelings indirectly and reduce emotional confrontation. Finally, the free drawing stage provides a safe, private space for unrestricted emotional expression, promoting emotional exploration and self-reflection without fear of judgment. These stages work together to offer students an intuitive, nonverbal way to engage with and understand their emotions (Multimedia Appendix 2).

## Discussion

### Principal Findings

This study aimed to develop a coloring-based digital art therapy intervention for treating alexithymia among Chinese college students. Through expert interviews, we gathered valuable insights that informed the creation of the intervention framework. The findings emphasized the effectiveness of using color, symbolism, and nonverbal emotional expression methods to address the challenges faced by college students with emotional processing difficulties. These insights align with existing theories highlighting the importance of visual over verbal expression in facilitating emotional regulation and expression for individuals with alexithymia [43]. Based on this framework, the *Coloring the Emoji* game was developed as a therapeutic intervention that uses coloring tasks to enhance emotional recognition and expression, offering a nonverbal, intuitive platform for self-reflection.

### Intervention Framework

The intervention framework for coloring-based digital art therapy, developed through expert input, focuses on addressing the core features of alexithymia, specifically the difficulty in identifying and describing emotions. By integrating visual symbols such as colors and patterns, the framework provides a tangible method for Chinese college students to externalize their emotions, making it easier for them to communicate feelings they may struggle to verbalize [44,45]. Experts also emphasized the importance of creating a nonjudgmental and low-pressure environment for emotional expression. The design of the intervention explicitly avoids grading or labeling emotional outputs, ensuring that students can express themselves without the fear of judgment, which is particularly important for those with alexithymia who may already experience difficulties with emotional expression [46]. Furthermore, the integration of digital technology was deemed essential for creating a seamless, intuitive, and multisensory experience, allowing students to explore their emotions continuously and with reduced anxiety [47].

By combining expert insights, the intervention framework incorporates visual, symbolic, and contextual elements, making emotional exploration more relatable and personalized for students. The framework emphasizes incorporating body language and sensory feedback through adjustments to brush size and intensity, further enhancing the emotional expression process and providing a more immersive and realistic experience [42]. These principles were then directly applied in the development of *Coloring the Emoji*, creating a user-friendly, digital intervention that meets the therapeutic goals outlined in the framework.

### Coloring Game

In the design of *Coloring the Emoji*, several key features were incorporated based on expert feedback to specifically address the emotional needs of Chinese college students with alexithymia. The color selection phase draws upon emotional psychology models, simplifying complex emotions into 2 dimensions—pleasure and arousal—and mapping these to specific colors [48]. This approach helps users quickly identify and select colors that best represent their emotional state, facilitating easier emotional expression. The pattern selection phase further supports emotional identification by offering a library of emojis, which were carefully selected for their strong emotional polarity [49]. Emojis are widely used in Chinese digital communication, providing students with a familiar and culturally relevant means to connect emotions with symbolic representations, thus making the emotional expression process more relatable.

The free drawing phase allows for uninhibited emotional expression, reinforcing the importance of a nonjudgmental space for self-reflection. The inclusion of interactive options, such as brush resizing and adjusting movement intensity, enhances user engagement and allows students to express their emotions more freely [50]. These features also align with expert recommendations for integrating sensory feedback into the therapeutic process, providing a more interactive and immersive emotional exploration experience. Additionally, the game’s structure supports nonverbal emotional expression, which is particularly beneficial for Chinese students, who may face cultural challenges in verbalizing their emotions.

The game also incorporates robust privacy protection measures to ensure the confidentiality of students’ emotional data [51]. The option to share their artwork anonymously supports a safe, private space for emotional expression, reducing the social pressure that might otherwise inhibit participation. These privacy features, combined with the therapeutic design elements, ensure that students can engage with the game in a way that feels secure and supportive, while respecting their cultural values and emotional boundaries [52].

### Limitations and Future Directions

While *Coloring the Emoji* shows promise in supporting emotional expression and regulation for Chinese college students with alexithymia, several limitations remain that need to be addressed in future research. This study focused specifically on Chinese college students, whose emotional challenges are influenced by cultural factors such as emotional restraint and social conformity. While the game design addresses these cultural nuances, its applicability to other populations remains uncertain. Future studies should explore how the game can be adapted to meet the needs of different cultural and age groups, as well as individuals with varying emotional processing difficulties. This would involve adjusting the game to accommodate diverse emotional challenges in different contexts, ensuring that the intervention remains relevant and effective across various demographics.

The current prototype requires rigorous user testing to assess its therapeutic impact and identify areas for improvement. Although expert feedback has been crucial in shaping the design, real-world testing is essential to evaluate how well the game supports emotional regulation and expression in practical settings [53,54]. Additionally, continuous collection of user data and qualitative feedback will provide insights into the game’s effectiveness and adaptability to users’ specific emotional needs.

Although the *Coloring the Emoji* game is beneficial for individuals with alexithymia, it may not fully replace other therapeutic methods, such as CBT or traditional art therapy [55,56]. Future research could investigate the potential integration of this coloring-based intervention with established therapeutic methods, creating a more comprehensive treatment plan. Combining digital art therapy with conventional therapies could offer a more holistic and adaptable model for treating emotional processing difficulties [57].

### Conclusions

This study proposes a coloring-based digital art therapy framework developed to address alexithymia among Chinese college students. Based on this framework, we created *Coloring the Emoji*, a platform that provides an intuitive, nonverbal, and low cognitive-load method for emotional identification and expression. By integrating color, symbolism, and sensory feedback, the game offers a nonjudgmental space for anonymous self-expression, which directly addresses the cultural norms of emotional restraint and societal barriers faced by this population. The inclusion of emojis, which are widely popular in Chinese digital communication, further assists students in connecting emotions with familiar symbols. Additionally, the platform is free of charge and easily accessible, making it suitable for students facing academic pressures and financial constraints.

While the intervention shows promise, further research is needed to assess its real-world effectiveness and adaptability across diverse cultural and age groups. Future studies could explore the integration of *Coloring the Emoji* with other therapeutic approaches, such as CBT, to enhance its therapeutic impact. Further research should also examine its long-term effects, particularly in addressing co-occurring emotional issues, such as anxiety and stress.

## Supplementary material

10.2196/82128 Multimedia Appendix 1 Interview guide.

10.2196/82128 Multimedia Appendix 2 Description of the user task of *Coloring the Emoji*.

## Abbreviations

CBT: cognitive behavioral therapy
