# Supplementary material for: Designing Coloring-Based Digital Art Therapy to Treat Alexithymia in Chinese College Students: Qualitative Study
Source: JMIR Hum Factors. 2026 Jun 1;13:e82128. doi: 10.2196/82128 (PMC13225227; doi:10.2196/82128)
Supplement: Multimedia Appendix 1 [file humanfactors-v13-e82128-s001.docx]

## Multimedia Appendix 1

**Interviewer instructions:** To gather expert perspectives on developing a coloring-based digital art therapy framework for Chinese college students with alexithymia, considering the cultural and emotional challenges specific to this demographic.

Approach:

1. Use open-ended questions.
2. Prioritize depth over breadth.
3. Allow skip/pause at any time.
4. Duration: 60 minutes.

**Introduction**

Thank you for joining us. We’re exploring a digital coloring intervention to help Chinese college students who struggle to identify emotions (alexithymia). In China, emotional restraint is culturally valued, which can make emotional expression particularly challenging for college students. Your expertise will guide our framework. This interview will take about an hour. May I record our conversation? (Introduce interviewers)

**About you**

- Could you briefly share your background (Age, Professional title, educational background, etc.)?
- How long have you worked in emotion-related fields, particularly in working with Chinese college students?

**Art Therapy Experiences**

- Have you used art-based approaches before?
- Could you describe 1-2 memorable experiences with Chinese college students?
- What specific tools or methods did you use?
- Which of these tools/methods were effective in engaging Chinese college students?
- Which didn’t work as expected? What challenges did Chinese college students face during activities?
- When Chinese college students avoided discussing feelings, how did art help?

**Attitude to Digital Art Therapy**

- What’s your view on using digital art therapy for emotion regulation of Chinese college students?
- Why do you hold this attitude?
  - Prompts: Positive? Negative? Neutral?
- Specifically for coloring games: What would make them truly helpful for this demographic?

**Suggestions for Coloring-based Serious Games**

- What three rules would you insist on for this kind of game?
- What one thing would you absolutely avoid?
- How should we adapt this for Chinese college students?
- What other suggestions do you have for designing coloring games?

**Willingness of the expert to facilitate and support the follow-up study**

- Would you like to review our work later?
- Could you suggest other experts we should contact?

**Conclusion**

I truly value you taking the time to share these insights with us today. Your expertise is invaluable as we develop this intervention. Sometimes new perspectives emerge after reflection, if any additional thoughts come to mind in the next day or two, please feel welcome to reach out to us, and we welcome your follow-up at any time.
