# Supplementary material for: Designing Coloring-Based Digital Art Therapy to Treat Alexithymia in Chinese College Students: Qualitative Study
Source: JMIR Hum Factors. 2026 Jun 1;13:e82128. doi: 10.2196/82128 (PMC13225227; doi:10.2196/82128)
Supplement: Multimedia Appendix 2 [file humanfactors-v13-e82128-s002.docx]

## Multimedia Appendix 2

*Coloring the Emoji* is an interactive digital art therapy game designed to support Chinese college students in emotional expression and regulation. The game offers an intuitive, non-verbal platform where students can process their emotions through color selection, pattern selection, and free coloring. The overall process is shown in the **User Manual** (Figure 1).

In detail, the game begins with a **Start Page** (Figure 1), where students can press the "Start" button to begin their emotional exploration. The **Color Selection Page** (Figure 2) helps students utilize the *Emotion Color Gear* to identify and externalize their emotions by linking them with specific colors. The **Pattern Selection Page** (Figure 3) features the *Emoji Sense Library*, enabling students to connect their emotions with real-life situations using familiar symbols, which enhances emotional understanding. The **Free Coloring Page** (Figure 4) provides a space for students to freely express their emotions through coloring, fostering creativity and emotional release. After customizing their emoji, students can preview their work in a **Secondary Preview Window** (Figure 5), with options to save or upload their creation. The **History Page** (Figure 6) showcases a collection of pre-colored works, allowing students to evaluate their emotional state.

Below, we outline the detailed functions of *Coloring the Emoji*, including its core design principles and how each feature supports emotional processing.


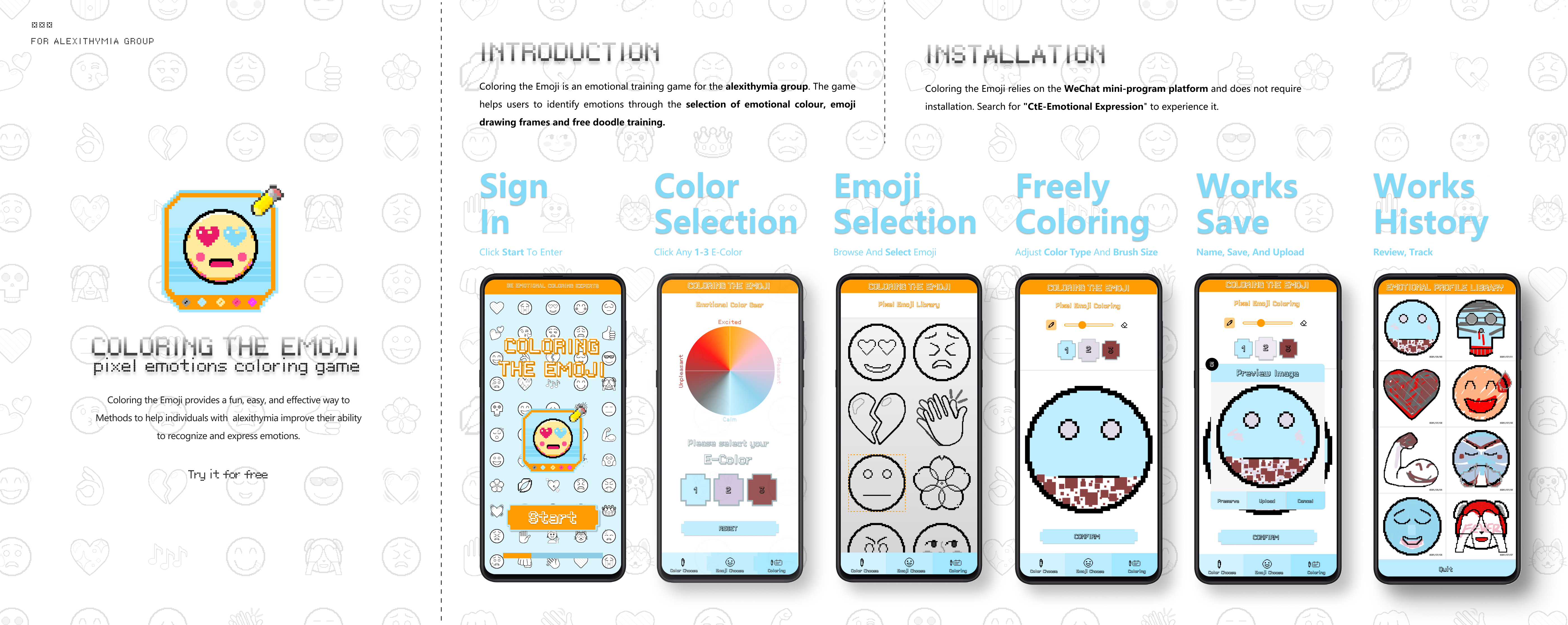


Figure 1. **User Manual.**





Figure 1. **Start Page.**

Users can press the Start button to start the emotional exploration process. The background screen is composed of a series of emoticons, with blue as the main color and orange as the accent color, creating a relaxed and warm gaming environment.





Figure 2. **Color Selection Page.**

Users tap the emotional color gear to select a color that represents their feelings, beginning their emotional expression journey. This step features undo and redo options for easy adjustment. Users can select up to three emotional colors, with the selected colors displayed in the three boxes below. A navigation bar at the bottom guides users through the three main steps—color selection, emoji selection, and free coloring—allowing them to adjust their actions at any time.





Figure 3. **Pattern Selection Page.**

Users can select a pixel-style emoji that best represents their current emotional state. A variety of emojis (including facial expressions, gestures, and symbols) are arranged according to emotional polarity, from pleasant to unpleasant, making it easy for users to intuitively identify emotions.





Figure 4. **Free Coloring Page.**

Users can color emojis using their chosen emotional colors. The top area contains a brush size slider for fine-grained control, while a live coloring area in the center of the screen displays the user's coloring progress. A "Confirm" button at the bottom confirms the completion of the free coloring stage and directly jumps to the Secondary Preview Window.





Figure 5. **Secondary Preview Window.**

Users can view their own works and are provided with three options: Save the work to the history; upload the work to the sharing community; or cancel the work review and return to the previous step.

`



Figure 6. **History Page.**

Users can review their past creations. Each coloring work is timestamped to show when it was created, allowing users to track their emotional expressions over time. This history provides a simple and personalized way to relive past emotions and creative moments.
